# The effect of Astragalus polysaccharides on attenuation of diabetic cardiomyopathy through inhibiting the extrinsic and intrinsic apoptotic pathways in high glucose -stimulated H9C2 cells

**DOI:** 10.1186/s12906-017-1828-7

**Published:** 2017-06-13

**Authors:** Shuqin Sun, Shuo Yang, Min Dai, Xiujuan Jia, Qiyan Wang, Zheng Zhang, Yongjun Mao

**Affiliations:** 1 Department of Geriatrics, the Affiliated Hospital of Qingdao University, Qingdao, 266003 China; 2 Department of the Intensive Care Unit, the Affiliated Hospital of Qingdao University, Qingdao, 266003 China; 3 School of Life Sciences, Beijing University of Chinese Medicine, Beijing, 100029 China

**Keywords:** Diabetic cardiomyopathy, Apoptosis, The extrinsic pathway, The intrinsic pathway, Astragalus polysaccharides

## Abstract

**Background:**

Apoptosis plays a critical role in the progression of diabetic cardiomyopathy (DC). Astragalus polysaccharides (APS), an extract of *astragalus membranaceus* (AM), is an effective cardioprotectant. Currently, little is known about the detailed mechanisms underlying cardioprotective effects of APS. The aims of this study were to investigate the potential effects and mechanisms of APS on apoptosis employing a model of high glucose induction of apoptosis in H9C2 cells.

**Methods:**

A model of high glucose induction of H9C2 cell apoptosis was adopted in this research. The cell viabilities were analyzed by MTT assay, and the apoptotic response was quantified by flow cytometry. The expression levels of the apoptosis related proteins were determined by Real-time PCR and western blotting.

**Results:**

Incubation of H9C2 cells with various concentrations of glucose (i.e., 5.5, 12.5, 25, 33 and 44 mmol/L) for 24 h revealed that cell viability was reduced by high glucose dose-dependently. Pretreatment of cells with APS could inhibit high glucose-induced H9C2 cell apoptosis by decreasing the expressions of caspases and the release of cytochrome C from mitochondria to cytoplasm. Further experiments also showed that APS could modulate the ratio of Bcl-2 to Bax in mitochondria.

**Conclusions:**

APS decreases high glucose-induced H9C2 cell apoptosis by inhibiting the expression of pro-apoptotic proteins of both the extrinsic and intrinsic pathways and modulating the ratio of Bcl-2 to Bax in mitochondria.

## Background

Diabetes mellitus is one of the major causes of mortality and morbidity worldwide. The number of adult diabetic patients will continue to increase globally due to an aging population, growth of population size, urbanization and high prevalence of obesity [1]. Among the complications, diabetic cardiomyopathy (DC) is the leading cause of death for diabetic patients. Diabetic cardiomyopathy is first described by Rubler in 1972 as cardiac structural abnormalities in the absence of hypertension, coronary heart diseases and other specific traditional diseases [2]. The early manifestation of DC is diastolic dysfunction, followed by the systolic dysfunction and eventual heart failure [3]. The pathophysiology of diabetic cardiomyopathy is not completely understood. DC could be caused by the interactions among multiple factors such as hyperglycemia, hyperinsulinemia/hypoinsulinemia, abnormal fatty acid metabolism, oxidative stress and cardiac autonomic neuropathy [4]. Recent studies showed that there was an increase of cellular apoptosis in the heart of diabetic patients, Streptozocin (STZ)-induced diabetic animals and high glucose-treated cardiomyocytes [5–7]. Apoptosis is an important contributing factor to pathology in diabetes mellitus. Apoptosis leads to cardiac cell loss, decreased cardiac contractile ability and eventually cardiac remodeling [8]. The extent of cardiomyocyte death parallels the severity of the diabetic cardiomyopathy and its stage of evolution [7]. Attenuation of cardiomyocyte apoptosis has been shown to prevent diabetic cardiomyopathy [8, 9]. The mechanisms of apoptosis involved in diabetic cardiomyopathy are not clear. Studies suggest that it may be related to oxidative/nitrative stress and increased production of inflammatory factors such as TNF-α [10–12].

Caspases are inactive in the cells and can be activated when cleaved upon induction of the apoptotic program [13]. During diabetic cardiomyopathy, cardiomyocyte apoptosis is initiated through intrinsic pathway (the mitochondria-mediated pathway) and extrinsic pathway (the death receptor-mediated pathway) [10, 14]. Either activated caspase-8 in the extrinsic pathway or cytochrome C activated caspase-9 in the intrinsic pathway could activate the final executor caspase-3 to result in apoptosis [13].

Mitochondrial membrane integrity is a key protecting factor against apoptosis. Bcl-2 family acts at the outer mitochondrial membrane and is involved in regulating mitochondrial membrane integrity, whereas the pro-apoptotic factor Bax is involved in the intrinsic pathway controlling apoptosis [15]. When activated, Bax stimulates the release of cytochrome C and other apoptogenic mitochondrial proteins into the cytosol to trigger apoptosis. On the other hand, Bcl-2 antagonizes this process. It is hypothesized that the cell fate is determined by the ratio of Bcl-2 to Bax [16]. Drugs that can interfere with any part of the intrinsic or extrinsic pathway or the Bcl-2 family can reduce apoptosis in diabetic cardiomyopathy [17]. Therefore, factors in the intrinsic or extrinsic apoptotic pathway or the Bcl-2 family can be potential therapeutic targets for diabetic cardiomyopathy.

Many traditional Chinese herbs have been used for treating diabetes, among which the root of *Astragalus membranaceus* (AM, Huangqi) is one notable that has been used for thousands of years. AM contains 68 components such as polysaccharides, saponins, flavonoids, etc. Astragalus polysaccharides (APS) has been identified as the major ingredient with anti-diabetic activity [18]. More and more evidence shows that APS can lower blood glucose and lipid levels, and improve insulin resistance in diabetic rats [18, 19]. APS also plays an important role in attenuating diabetic complications. Wang et al. suggested that APS could alleviate liver endoplasmic reticulum stress both in high glucose induced hepatocytes and in type 2 diabetes mellitus (T2DM) rats [20]. Zhang et al. showed that APS could improve early diabetic nephropathy through modulating mRNA expressions of nuclear factor kappa B (NF-κB) and inhibitor kappa B (IκB) in renal cortex of diabetic rats [21]. For diabetic cardiomyopathy, Chen et al. suggested that APS could modulate glucose and lipids metabolism through peroxisome proliferator activated receptor (PPAR)-α pathway and reduce cardiac fibrosis through suppression of local cardiac chymase-Angiotensin II system [22, 23]. However, the effects and molecular mechanisms of APS on the main pathological change of diabetic cardiomyopathy are far from clear. The objective of this study was to investigate the inhibition of APS on cardiac apoptosis in high glucose-stimulated H9C2 cells. The underlying molecular mechanisms of APS on the cardiomyocytes were also investigated in this study.

## Methods

### Cell culture and treatments

H9C2 cells were purchased from the Shanghai Cellular Research Institute (Shanghai, China) and cultured in Dulbecco’s modified essential medium (DMEM, Hyclone, USA) supplemented with 10% fetal bovine serum (FBS, Hyclone, USA) and 1% penicillin-streptomycin (Hyclone, USA) at 37 °C in an incubator with a humidified atmosphere of 5% CO_2_. During the experimental period, cells were divided into several groups, which were treated with low glucose, high glucose and high glucose combined with APS, respectively. The APS group was pretreated with APS for 24 h, followed by incubation with high glucose for an additional 24 h. APS was purchased from Tianjin Cinorch Pharmaceutical Company as a hazel-colored and water-soluble powder. The minimum purity was determined to be 98%. The APS consists of α-1,4 (1,6) dextrans, arabinogalactan (AGs), rhamnogalacturonan I (RGIs) and arabinogalactan-proteins (AGPs) compositions, and it has a molecular weight between 40,000–80,000. Its monosaccharide composition is mainly composed of glucose, arabinose, galactose, rhamnose and galacturonic acid, among which glucose, arabinose and galactose are the main components, accounting for more than 90% of the whole composition.

### Determination of cell viability

Cell viability was measured using the 3-(4,5-dimethylthiazol-2-yl)-2,5-diphenyl-tetrazolium bromide (MTT) assay (Sigma, USA). Briefly, The H9C2 cardiomyocytes were cultured at a density of 1 × 10^5^ cells/ml in 96-well plates and divided into groups of normal glucose (5.5 mmol/L), high glucose (12.5, 25, 33, 44 mmol/L) and high glucose combined with different concentrations of APS (0.1, 0.2, 0.4, 0.8, 1.6 and 3.2 mg/mL) for 24 h. Then 20 μL MTT was added (5 mg/mL final concentration in medium) and the H9C2 cardiomyocytes were incubated for another 4 h at 37 °C. The medium was then abandoned and 150 μL dimethyl sulfoxide (DMSO) was added to each well which was then shaken for 10 min at room temperature to completely dissolve the blue-purple precipitate from the MTT. The absorbance was measured at 490 nm using a Microplate Reader (Thermo, USA). The absorbance of 6 wells for each group was averaged. To exclude the hyperosmolar effect of high glucose on H9C2 cell viability, identical concentration of mannitol in normal glucose group was added.

### Flow cytometry analysis of apoptotic cells

The apoptotic cells were detected by flow cytometry by means of annexin V-FITC/propidium iodide (PI) staining (BD, USA) following the manufacturer’s instructions. After treatment for 24 h, the cells were harvested and washed in cold phosphate buffer saline (PBS) twice, then double stained by Annexin V and PI in 100 μL 1 × binding buffer at room temperature for 15 min in the dark. After incubation, another 400 μL 1× annexin-binding buffer was added. The apoptotic cells were then analyzed by a flow cytometry (BD, USA).

### Quantitative real-time polymerase chain reaction (PCR)

Total RNA was extracted from H9C2 cells using 1 mL TRIzol reagent (Invitrogen, Carlsbad, CA) following the manufacturer’s instructions. All RNA samples were kept at −80 °C until use. The PCR primer sequences were designed according to the gene sequences reported in GenBank and were chemically synthesized. Each sample was tested in triplicate, with the use of the quantitative SYBR Green PCR kit (VAZYME, Nanjing, China) for 40 cycles (50 °C for 2 min, 95 °C for 10 min, 95 °C for 30 s, and 60 °C for 30 s) on the ABI 7900HT fast real time PCR System (Applied Biosystems, Foster, CA). The primers were as follows: GAPDH forward primer, ACAGCAACAGGGTGGTGGAC, reverse primer, TTTGAGGGTGCAGCGAACTT; Bax forward primer, TGGCGATGAACTGGACAAC, reverse primer, GCAAAGTAGAAAAGGGCAACC; Bcl-2 forward primer, CCTGGCATCTTCTCCTTCCA, reverse primer, GGACATCTCTGCAAAGTCGC; caspase 3 forward primer, GGACCTGTGGACCTGAAAAA, reverse primer, GCATGCCATATCATCGTCAG; caspase 8 forward primer, TTCTGTTTTGGATGAGGTG, reverse primer, TTGCTGAGTTTGGGTATGT; caspase 9 forward primer, CACTGCCTCATCATCAACAAC, reverse primer, TGTGCCATAGACAGCACCC; cytochrome C forward primer, TGTTCAAAAGTGTGCCCAGT, reverse primer, CTTCTTCTTAATTCCAGCG. Cycle threshold (Ct) values were obtained graphically for the target genes and GAPDH. The relative fold change in gene expression was calculated as 2-ΔΔCt.

### Western blotting

The extraction of cell cytoplasmic and mitochondrial fractions were performed using the Mitochondria/Cytosol Fractionation Kit (BioVision, CA, USA) according to the manufacturer’s protocol. Total cell proteins were extracted by RIPA lysing buffer (Beyotime Institute of Biotechnology, China) and protein concentration was determined by the BCA Bradford protein assay kit (Beyotime Institute of Biotechnology, China). The extracted proteins were then mixed with loading buffer and boiled for 10 min. Equal amounts of proteins (40 μg) were separated using sodium dodecyl sulfate-polyacrylamide gel electrophoresis (SDS-PAGE) and transferred to polyvinylidene difluoride (PVDF) membranes (Millipore, Germany). After being blocked with 5% non-fat dry milk for 2 h, membranes were incubated overnight at 4 °C with primary antibodies against GAPDH (1:1000, Abcam, USA), VDAC (1:2000, abcam, USA), cytochrome C (1:500, Abcam, USA), Bcl-2 (1:500, Abcam, USA), Bax (1:800, Abcam, USA), cleaved caspase 3 (1:500, Abcam, USA), caspase 8 (1:500, Abcam, USA), cleaved caspase 9 (1:500, Abcam, USA), and then incubated with horseradish peroxidase-conjugated secondary antibody (ZSGB-BIO, China) for 2 h at room temperature. Membranes were visualized by an enhanced chemiluminescence (ECL) kit (Thermo, USA) and quantified by densitometry using an image analyzer (BandScan, USA).

### Statistical analysis

Statistical analysis was performed using one-way analysis of variance (ANOVA) and least significant difference (LSD) post hoc analysis by using SPSS 17.0 (SPSS Inc., Chicago, USA). All data are presented as Mean ± standard deviation (SD). *P* < 0.05 were considered significant.

## Results

### High glucose dose-dependently induced apoptosis in H9C2 cells

**Fig. 1:**
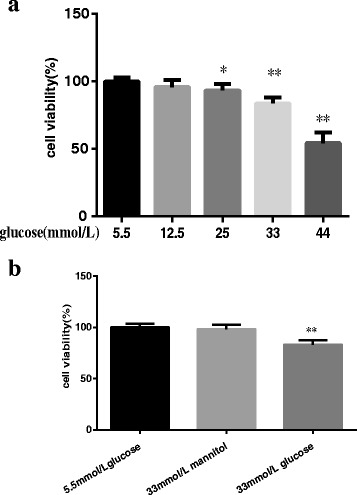
**a** Cell viabilities in cells treated with various concentrations of glucose for 24 h. MTT assay was used to measure cell viability. Data were expressed as Mean ± SD. ^*^ *P* < 0.05 versus control. ^**^ *P* < 0.01 versus control. **b**. Cell viabilities in cells treated with 5.5 mmol/L glucose group, 5.5 mmol/L glucose with 33 mmol/L mannitol and 33 mmol/L glucose groups for 24 h. MTT assay was used to measure cell viability. Data were expressed as Mean ± SD. ^**^ *P* < 0.01 versus control

H9C2 cells were incubated with various concentrations of glucose (5.5, 12.5, 25, 33 and 44 mmol/L) for 24 h and cell viabilities were measured by MTT assay. The results revealed that cell viabilities were not significantly different between 5.5 mmol/L and 12.5 mmol/L glucose-treated groups. However, as the glucose concentration increased, the cell viabilities decreased dose-dependently. Compared with the 5.5 mmol/L-treated control group, the cell viabilities in the 33 mmol/L and 44 mmol/L glucose-treated group decreased significantly, by nearly 16% and 45%, respectively (100.00 ± 3.05 vs 83.77 ± 4.43; 100.00 ± 3.05 vs 54.37 ± 7.79; *P* < 0.01. Fig. 1a). As 33 mmol/L glucose could mimic hyperglycemia in vivo, this concentration was selected for the following experiment. To further exclude the osmotic effect of high glucose on H9C2 cell viability, the cell viabilities of 5.5 mmol/L glucose group, 5.5 mmol/L glucose with 33 mmol/L mannitol and 33 mmol/L glucose groups were compared. Mannitol had no effect on cell viability (100 ± 3.85 vs 98.25 ± 4.45; *P* > 0.05. Fig. 1b), indicating that hyperosmolar effect could be excluded for high glucose-induced cell viability decrease. Based on these data, 33 mmol/L was chosen for the further experiments.

### Effects of APS on cell viabilities of high glucose-treated H9C2 cells

**Fig. 2:**
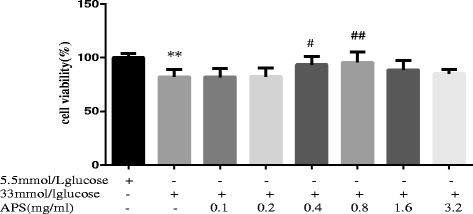
Cell viabilities in cells treated with 5.5 mmol/L glucose, 33 mmol/L glucose or 33 mmol/L glucose in combination with different concentrations of APS (0.1, 0.2, 0.4, 0.8, 1.6 and 3.2 mg/mL) for 24 h. MTT assay was used to measure cell viability. Data were expressed as Mean ± SD. ^**^ *P* < 0.01 versus control. ^#^ *P* < 0.05 versus 33 mmol/L glucose group. ^##^ *P* < 0.01 versus 33 mmol/L glucose group

In the APS treated groups, H9C2 cells were pretreated with different concentrations of APS (0.1, 0.2, 0.4, 0.8, 1.6 and 3.2 mg/mL) for 24 h and then incubated with high glucose (33 mmol/L) for 24 h. The control group and high glucose group were incubated with 5.5 mmol/L and 33 mmol/L of glucose for 24 h, respectively. The cell viabilities were measured by MTT assays. The results showed that high glucose could significantly decrease cell viabilities (100.00 ± 3.98 vs 82.2 ± 6.85; *P* < 0.01, Fig. 2). Compared to cells treated with high glucose alone, pretreatment with 0.4 mg/mL or 0.8 mg/mL APS could increase cell viabilities, and cells pretreated with 0.8 mg/mL APS had the highest cell viabilities (82.2 ± 6.85 vs 95.70 ± 9.75; *P* < 0.01, Fig. 2). Therefore, the 0.8 mg/mL APS was selected for the following experiments.

### APS protected H9C2 cells from high glucose-induced apoptosis

**Fig. 3:**
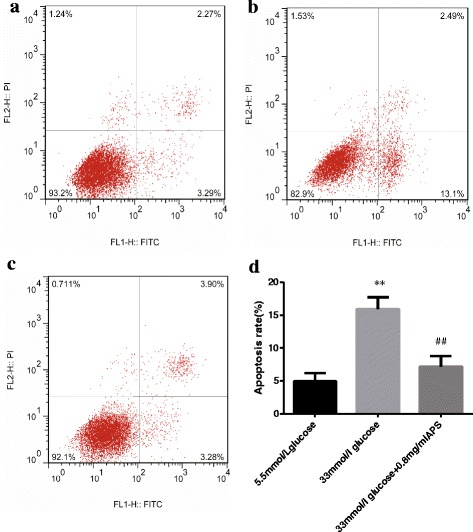
Cell apoptotic rates measured by flow cytometry using annexin V-FITC/PI staining. H9C2 cells were pre-incubated with/without APS for 24 h and then treated with 33 mmol/L glucose for 24 h. Cells were harvested and detected with a flow cytometer. **a**. 5.5 mmol/L glucose group. **b**. 33 mmol/L glucose group. **c**. 33 mmol/L glucose and 0.8 mg/mL APS group. Data were expressed as Mean ± SD. ^**^ *P* < 0.01 versus 5.5 mmol/L glucose group; ^##^ *P* < 0.01 versus 33 mmol/L glucose group

To further elucidate whether APS could inhibit high glucose-induced H9C2 cell apoptosis, annexin V/PI double labeling flow cytometric analysis was conducted to determine cell apoptosis rates in each group. As shown in Fig. 3, the apoptotic rates were significantly higher in high glucose group compared with that of low glucose (4.92 ± 1.27 vs 15.96 ± 1.81; *P* < 0.01, Fig. 3). The apoptotic rate of cells treated with high glucose and APS decreased significantly as compared with that of cells treated with high glucose alone (15.96 ± 1.81 vs 7.20 ± 1.60; *P* < 0.01, Fig. 3), demonstrating that APS could attenuate apoptosis induced by high glucose in H9C2 cells.

### Effects of APS on the mRNA expression levels of extrinsic apoptotic pathway-related factors

**Fig. 4:**
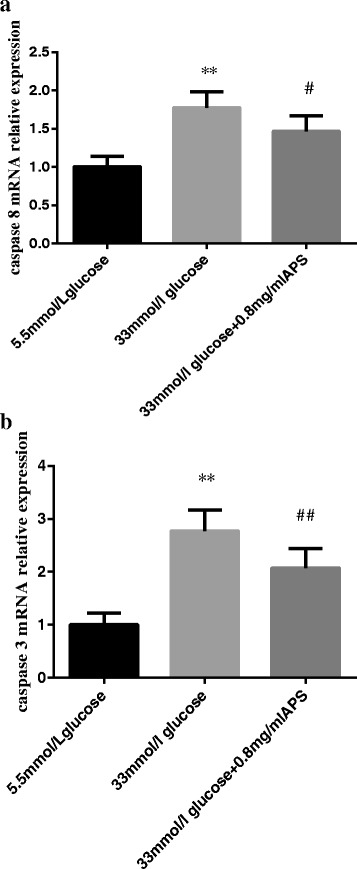
APS modulated the mRNA expression levels of extrinsic apoptotic pathway-related factors. H9C2 cells were pre-incubated with/without APS for 24 h and then treated with 33 mmol/L glucose for 24 h. The expression of GAPDH was measured as an internal control. **a**. the relative mRNA expression levels of caspase 8 in different groups. **b**. the relative mRNA expression levels of caspase 3 in different groups. Data were expressed as Mean ± SD. ^**^ *P* < 0.01 versus 5.5 mmol/L glucose group; ^#^ *P* < 0.05 versus 33 mmol/L glucose group. ^##^ *P* < 0.01 versus 33 mmol/L glucose group

To investigate whether APS could regulate the extrinsic apoptotic pathway-related factors, the mRNA expression levels of caspase 8 and caspase 3 were determined by quantitative real-time PCR analysis. In comparison to the low glucose group, high glucose treatment increased the mRNA expression levels of caspase 8 and caspase 3 (caspase 8 1.00 ± 0.14 vs 1.77 ± 0.21; caspase 3 1.00 ± 0.22 vs 2.78 ± 0.39; *P* < 0.01, Fig. 4a, b). Pretreatment with APS could remarkably inhibit the expression levels of caspase 8 and caspase 3 (caspase 8 1.77 ± 0.21 vs 1.46 ± 0.21, *P* < 0.05; caspase 3 2.78 ± 0.39 vs 2.07 ± 0.37; *P* < 0.01, Fig. 4a, b).

### Effects of APS on the mRNA expression levels of intrinsic apoptotic pathway-related factors

**Fig. 5:**
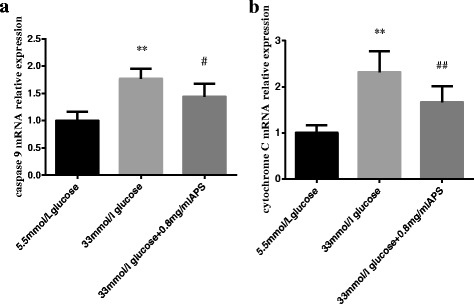
APS modulated the mRNA expression levels of intrinsic apoptotic pathway-related factors. H9C2 cells were pre-incubated with/without APS for 24 h and then treated with 33 mmol/L glucose for 24 h. The expression of GAPDH was measured as an internal control. **a**. the relative mRNA expression levels of caspase 9 in different groups. **b**. the relative mRNA expression levels of cytochrome C in different groups. Data were expressed as Mean ± SD. ^**^ *P* < 0.01 versus 5.5 mmol/L glucose group; ^#^ *P* < 0.05 versus 33 mmol/L glucose group. ^##^ *P* < 0.01 versus 33 mmol/L glucose group

To determine whether APS could suppress the intrinsic apoptotic pathway in high glucose stimulated H9C2 cells, the mRNA expression levels of caspase 9 and cytochrome C were measured. The results showed that the levels of caspase 9 and cytochrome C were up-regulated in high glucose group (caspase 9 1.00 ± 0.16 vs 1.76 ± 0.18; cytochrome C 1.00 ± 0.17 vs 2.32 ± 0.45; *P* < 0.01, Fig. 5a, b) and APS could down-regulate them toward control levels (caspase 9 1.76 ± 0.18 vs 1.44 ± 0.23, *P* < 0.05; cytochrome C 2.32 ± 0.45 vs 1.66 ± 0.34; *P* < 0.01, Fig. 5a, b). These results indicated that the induction of intrinsic apoptotic pathway in high glucose group could be suppressed by APS pretreatment.

### Effects of APS on the mRNA expression levels of Bax and Bcl-2

**Fig. 6:**
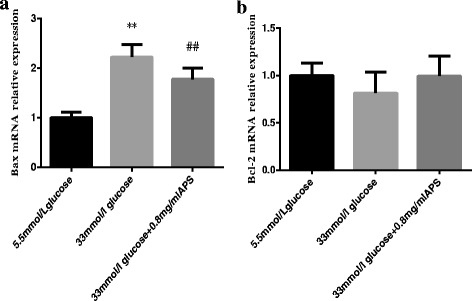
APS modulated the mRNA expression levels of Bax and Bcl-2. H9C2 cells were pre-incubated with/without APS for 24 h and then treated with 33 mmol/L glucose for 24 h. The expression of GAPDH was measured as an internal control. **a**. the relative mRNA expression levels of Bax in different groups. **b**. the relative mRNA expression levels of Bcl-2 in different groups. Data were expressed as Mean ± SD. ^**^ *P* < 0.01 versus 5.5 mmol/L glucose group; ^##^ *P* < 0.01 versus 33 mmol/L glucose group

Bcl-2 family was served as vital regulators of the intrinsic apoptotic pathway. To investigate whether APS could regulate the Bcl-2 family Bax and Bcl-2 mRNA, quantitative real-time PCR was used to quantity the levels of these mRNA. As shown in Fig. 6, high glucose could increase Bax mRNA expression level but had no significant effects on the Bcl-2 mRNA expression (Bax 1.00 ± 0.11 vs 2.23 ± 0.25; *P* < 0.01; Bcl-2 1.00 ± 0.13 vs 0.81 ± 0.22; *P* > 0.05, Fig. 6a, b). APS treatments resulted in a significant decrease of Bax mRNA expression level but had no significant effects on the Bcl-2 mRNA expression. (Bax 2.23 ± 0.25 vs 1.78 ± 0.23; *P* < 0.01; Bcl-2 0.81 ± 0.22 vs 1.00 ± 0.21, *P* > 0.05. Fig. 6a, b). The ratio of Bcl-2 to Bax was increased in APS treatment group as compared with that of the high glucose group.

### APS inhibited extrinsic apoptotic pathway in high glucose stimulated H9C2 cells

**Fig. 7:**
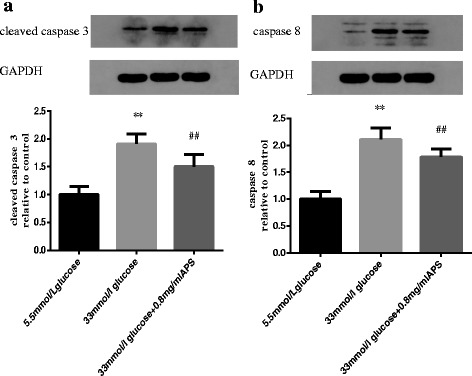
APS modulated apoptosis-related proteins in the extrinsic pathways. H9C2 cells were pre-incubated with/without APS for 24 h and then treated with 33 mmol/L glucose for 24 h. The expression of GAPDH was measured as an internal control for the cytoplasmic fraction. **a**. Semi-quantitative expressions of cleaved caspase 3 in different groups. **b**. Semi-quantitative expressions of caspase 8 in different groups. Data were expressed as Mean ± SD. ^**^ *P* < 0.01 versus 5.5 mmol/L glucose group; ^##^ *P* < 0.01 versus 33 mmol/L glucose group

To determine whether APS could suppress the extrinsic apoptotic pathway in high glucose stimulated H9C2 cells, the levels of cleaved caspase 3 and caspase 8 were measured. The results showed that the levels of cleaved caspase 3 and caspase 8 were up-regulated in high glucose group (cleaved caspase 3 1.00 ± 0.15 vs 1.91 ± 0.17; caspase 8 1.00 ± 0.14 vs 2.11 ± 0.22; *P* < 0.01, Fig. 7a, b) and APS could down-regulate them toward control levels (cleaved caspase 3 1.91 ± 0.17 vs 1.50 ± 0.22; caspase 8 2.11 ± 0.22 vs 1.79 ± 0.15; *P* < 0.01, Fig. 7a, b).

### APS inhibited intrinsic apoptotic pathway in high glucose stimulated H9C2 cells

**Fig. 8:**
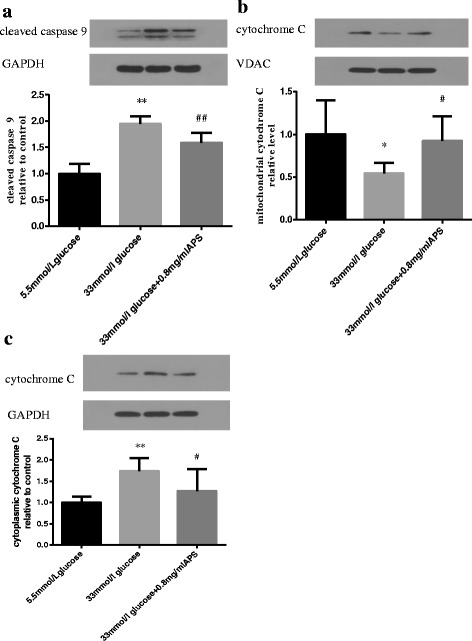
APS modulated apoptosis-related proteins in the intrinsic pathways. H9C2 cells were pre-incubated with/without APS for 24 h and then treated with 33 mmol/L glucose for 24 h. The expression of GAPDH was measured as an internal control for the cytoplasmic fraction. The expression of VDAC was measured as an internal control for the mitochondrial fraction. **a**. Semi-quantitative expressions of cleaved caspase 9 in different groups. **b**. Semi-quantitative expressions of mitochondrial cytochrome C in different groups. **c**. Semi-quantitative expressions of cytoplasmic cytochrome C in different groups. Data were expressed as Mean ± SD. ^*^ *P* < 0.05 versus 5.5 mmol/L glucose group; ^**^ *P* < 0.01 versus 5.5 mmol/L glucose group; ^#^ *P* < 0.05 versus 33 mmol/L glucose group; ^##^ *P* < 0.01 versus 33 mmol/L glucose group

Previous studies demonstrated that APS can attenuate high glucose-induced cellular apoptosis. To determine if APS could inhibit apoptosis through the intrinsic apoptotic pathway, the levels of cleaved caspase 9 and cytochrome C were studied. After cells were incubated with high glucose for 24 h, cleaved caspase 9 level was up-regulated (1.00 ± 0.18 vs 1.94 ± 0.15; *P* < 0.01, Fig. 8a) and release of cytochrome C from mitochondria to cytoplasm was increased (mitochondria cytochrome C 1.00 ± 0.40 vs 0.55 ± 0.12; *P* < 0.05. cytoplasm cytochrome C 1.00 ± 0.14 vs 1.74 ± 0.31; *P* < 0.01, respectively, Fig. 8b, c). These results indicated that the intrinsic apoptotic pathway was activated by high glucose. Pretreatment with APS could suppress these responses, suggesting that APS could inhibit cell apoptosis at least in part through suppression of the intrinsic apoptotic pathways (cleaved caspase 9 1.94 ± 0.15 vs 1.59 ± 0.19; *P* < 0.01. mitochondria cytochrome C 0.55 ± 0.12 vs 0.92 ± 0.29; *P* < 0.05. cytoplasm cytochrome C 1.74 ± 0.31 vs 1.27 ± 0.52; *P* < 0.05, respectively. Fig. 8a–c).

### APS could regulate mitochondrial levels of Bax and Bcl-2 in high glucose stimulated H9C2 cells

**Fig. 9:**
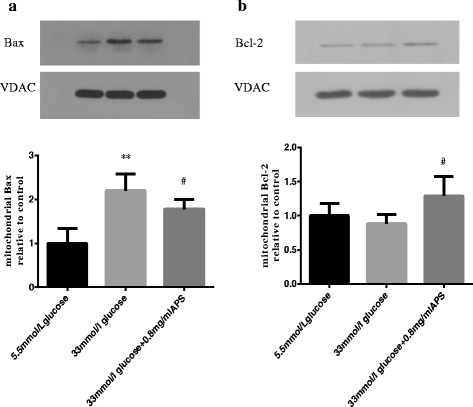
APS modulated apoptosis-related proteins of the Bcl-2 family. H9C2 cells were pre-incubated with/without APS for 24 h and then treated with 33 mmol/L glucose for 24 h. The expression of VDAC was measured as an internal control for the mitochondrial proteins. **a**. Semi-quantitative expressions of mitochondrial Bax in different groups. **b**. Semi-quantitative expressions of mitochondrial Bcl-2 in different groups. Data were expressed as Mean ± SD. ^**^ *P* < 0.01 versus 5.5 mmol/L glucose group; ^#^ *P* < 0.05 versus 33 mmol/L glucose group

The release of cytochrome C from mitochondria to cytoplasm is coordinated by the Bcl-2 family proteins, especially the ratio of Bcl-2 to Bax in mitochondria [16]. Therefore, the effects of APS on protein expressions of Bcl-2 and Bax were further investigated. The results showed that high glucose could increase Bax protein expression but had no significant effects on the Bcl-2 protein expression in the mitochondria (Bax 1.00 ± 0.34 vs 2.20 ± 0.38; *P* < 0.01. Bcl-2 1.00 ± 0.18 vs 0.88 ± 0.14; *P* > 0.05, respectively. Fig. 9a, b). The ratio of Bcl-2 to Bax decreased in high glucose group as compared to that of the control. APS treatments resulted in a significant decrease of Bax protein expression and a significant increase of Bcl-2 protein expression in mitochondria (Bax 2.20 ± 0.38 vs 1.78 ± 0.22; *P* < 0.05. Bcl-2 0.88 ± 0.14 vs 1.29 ± 0.29, *P* < 0.05. Fig. 9a, b) and the ratio of Bcl-2 to Bax increased in APS treatment group as compared with that of the high glucose group. These results indicated that APS could also suppress apoptosis through modulating the ratio of Bcl-2 to Bax.

## Discussion

Diabetic cardiomyopathy is the major complication of diabetes mellitus and the leading cause of death in diabetic patients. Accumulating evidences have proven that apoptosis plays a vital role in the pathophysiology of diabetic cardiomyopathy [5–9]. The use of plants and their derived substances has been increasing day by day owing to their versatile and powerful effects. For example, the seed extracts of *Syzygium fruticosum* Roxb and white mulberry possess significant antioxidant and anticancer properties [24, 25]. Polysaccharides present in multiple plants are constructed from monosaccharide unit and their derivatives, such as glucose, arabinose, galactose, rhamnose, etc. Polysaccharides isolated from *Salvia miltiorrhiza* showed cardioprotective effects against myocardial ischemia-reperfusion injury in rats by ameliorating oxidative stress and inhibiting myocardial apoptosis [26]. The polysaccharides could also be isolated from other plants such as *Lycium barbarum berries*, *Ganoderma atrum* and *Morchella importuna* [27–29]. APS is extracted from AM, a traditional Chinese herb that has been used in clinic for more than 3000 years. AM is distributed in Inner Mongolia, Shanxi, Gansu and other provinces of China. APS has been identified as the primary ingredient exerting immunomodulatory, anti-diabetic, anti-hypertrophic, anti-virus, anti-radiation activities [18, 30–32]. APS also plays an important role in attenuating diabetic complications such as diabetic cardiomyopathy and diabetic nephropathy, but the exact molecular mechanisms have remained unclear. In this study, the effects of APS on high glucose-induced apoptosis in H9C2 cells were explored and the anti-apoptotic mechanisms were investigated by MTT, annexin V-FITC/PI staining and western blotting methods. Our results showed that the anti-apoptotic function of APS was mediated through regulating the intrinsic/extrinsic apoptosis pathways and the Bcl-2 family.

Previous experiments showed that high glucose could induce apoptosis in H9C2 cells [33, 34]. In our study, consistent with previous observations, we found that high glucose could induce H9C2 cell apoptosis. Pretreatment with APS increased cell viability and reduced apoptotic rate in high glucose stimulated H9C2 cells, suggesting that APS possesses anti-apoptotic function in cardiomyocytes. The mechanisms by which APS exerted anti-apoptotic effects were further explored.

Various factors can activate caspases to initiate the apoptotic process. Apoptosis is mediated mainly by the extrinsic apoptotic pathway and the intrinsic apoptotic pathway [13, 35]. Extrinsic apoptosis is initiated through ligand-mediated activation of cell surface death receptors, such as the tumor necrosis factor receptors and CD95. Activated death receptors promote caspase-8 cleavage, which then activates down stream caspase 3. The intrinsic pathway starts from the release of cytochrome C from the intermembrane space of mitochondria into the cytosol. Cytochrome C, together with apoptosis activating factor 1 (Apaf-1), activate caspase 9, which in turn activates the executor caspase 3 [13]. It has been proven that during diabetes mellitus, high glucose can promote the release of cytochrome C into the cytoplasm and activate procaspase 3, which leads to the apoptosis of pancreatic islet cells by intrinsic pathway [36]. On the other hand, high glucose can promote the expression of Fas receptor in pancreatic islet cells, activate caspase 8 and increase the apoptosis of islet cells by extrinsic pathway [37]. In addition, the apoptotic rate of kidney and liver cells in the diabetic patients also increase significantly, which lead to the occurrence of diabetic nephropathy and diabetic liver disease [38, 39]. Previous studies have shown that APS could reduce apoptosis of beta cells by decreasing the expression of Fas [40]. Studies have also shown that APS could inhibit high fat diet induced apoptosis of H9C2 cells through lowering the expression of caspase 8 and caspase 3 and modulating the ratio of Bcl-2 to Bax [41]. These previous studies suggested that APS could inhibit apoptosis in islet and cardiac cells through multiple pathways. Our results showed that high glucose could induce apoptosis in H9C2 cells by promoting the release of cytochrome C into the cytoplasm, activating procaspase 9 and 3 and activating procaspase 8 and 3. After pretreatment of cells with APS, the release of cytochrome C into cytoplasm and expression of caspase 3, 8, 9 decreased as compared with those in high glucose treated group. Our results indicated that APS protected cells from apoptosis by modulating anti (pro)-apoptotic proteins of both intrinsic and extrinsic pathway.

Mitochondrial outer membrane permeabilization (MOMP) plays a key role in cell apoptosis [42, 43]. MOMP increases with apoptotic stimuli, which is followed by release of pro-apoptotic factors such as cytochrome C from mitochondria to cytoplasm to initiate intrinsic pathway of apoptosis. Bcl-2 family can modulate MOMP. This family includes both anti-apoptotic factors such as Bcl-2, Bcl-xL, and Mcl-1 and pro-apoptotic factors such as Bid, Bax and Bak. The pro-apoptotic family member Bax can undergo conformational change, and translocate from cytoplasm to the mitochondria when activated [44]. On the other hand, the anti-apoptotic family member Bcl-2 can antagonize this process. The ratio of Bcl-2 to Bax in mitochondria determines the cell fate [16, 45]. Studies showed that Bid deficiency or Bcl-2 overexpression could reduce the apoptosis of pancreatic islet cells induced by Fas or TNF-α. Loss of Bax or Bak could also reduce the apoptosis of pancreatic islet cells mediated by death receptors [46]. Other studies have also demonstrated that during diabetic nephropathy, the expression of Bcl-2 decreased and expressions of Bid and Bax increased. After treatment with mangiferin, the expression of Bcl-2 increased and expressions of Bid and Bax decreased [47]. These studies suggest that Bcl-2 family proteins are involved in the occurrence of apoptosis in diabetic nephropathy. APS has been shown to have anti-apoptotic function. Studies showed that APS could enhance the expression of Bcl-2, reduce the expression of caspase 3 and apoptosis of islet cells in type 1 diabetes mellitus [18]. Our experiment showed that high glucose could induce apoptosis in H9C2 cells by up-regulating Bax and reducing the ratio of Bcl-2 to Bax in mitochondria. After pretreatment of cells with APS, the expression of Bcl-2 in mitochondria increased, the expression of Bax in mitochondria decreased and the ratio of Bcl-2 to Bax increased. These results indicated that APS could inhibit high glucose induced apoptosis of H9C2 cells by modulating the ratio of Bcl-2 to Bax in mitochondria.

## Conclusions

The results of this study suggest that APS could attenuate cellular apoptosis induced by high glucose by inhibiting the expression of pro-apoptotic proteins of both the extrinsic and intrinsic pathway and modulating the ratio of Bcl-2 to Bax in mitochondria. The anti-apoptotic effect of APS confirms that it is an important therapeutic agent for the treatment of diabetic cardiomyopathy.

## Abbreviations

AGPs: Arabinogalactan-proteins
AGs: Arabinogalactan
AM: *Astragalus membranaceus*
ANOVA: Analysis of variance
Apaf-1: Apoptosis activating factor1
APS: Astragalus polysaccharides
Ct: Cycle threshold
DC: Diabetic cardiomyopathy
DMSO: Dimethyl sulfoxide
ECL: Enhanced chemiluminescence
IκB: Inhibitor kappa B
LSD: Least significant difference
MOMP: Mitochondrial outer membrane permeabilization
MTT: 3-(4,5-dimethylthiazol-2-yl)-2,5-diphenyl-tetrazolium bromide
NF-κB: Nuclear factor-kappa B
PBS: Phosphate buffer saline
PCR: Polymerase chain reaction
PI: Propidium iodide
PPAR: Peroxisome proliferator activated receptor
PVDF: Polyvinylidene difluoride
RGIs: Rhamnogalacturonan I
SD: Standard deviation
SDS-PAGE: Sodium dodecyl sulfate-polyacrylamide gel electrophoresis
STZ: Streptozocin
T2DM: Type 2 diabetes mellitus
